# The seroprevalence of SARS-CoV-2 during the first wave in Europe 2020: A systematic review

**DOI:** 10.1371/journal.pone.0250541

**Published:** 2021-11-02

**Authors:** Natasha Marcella Vaselli, Daniel Hungerford, Ben Shenton, Arwa Khashkhusha, Nigel A. Cunliffe, Neil French

**Affiliations:** 1 Institute of Infection, Veterinary & Ecological Sciences, University of Liverpool, Liverpool, United Kingdom; 2 NIHR HPRU in Gastrointestinal Infections at the University of Liverpool, Liverpool, United Kingdom; 3 NIHR HPRU in Emerging and Zoonotic Infections at the University of Liverpool, Liverpool, United Kingdom; 4 School of Medicine, Faculty of Life Sciences, University of Liverpool, Liverpool, United Kingdom; Technion - Israel Institute of Technology, ISRAEL

## Abstract

**Background:**

A year following the onset of the COVID-19 pandemic, new infections and deaths continue to increase in Europe. Serological studies, through providing evidence of past infection, can aid understanding of the population dynamics of SARS-CoV-2 infection.

**Objectives:**

This systematic review of SARS-CoV-2 seroprevalence studies in Europe was undertaken to inform public health strategies including vaccination, that aim to accelerate population immunity.

**Methods:**

We searched the databases Web of Science, MEDLINE, EMBASE, SCOPUS, Cochrane Database of Systematic Reviews and grey literature sources for studies reporting seroprevalence of SARS-CoV-2 antibodies in Europe published between 01/12/2019–30/09/20. We provide a narrative synthesis of included studies. Studies were categorized into subgroups including healthcare workers (HCWs), community, outbreaks, pregnancy and children/school. Due to heterogeneity in other subgroups, we only performed a random effects meta-analysis of the seroprevalence amongst HCWs stratified by their country.

**Results:**

115 studies were included spanning 17 European countries, that estimated the seroprevalence of SARS-CoV-2 from samples obtained between November 2019 –August 2020. A total of 54/115 studies included HCWs with a reported seroprevalence among HCWs ranging from 0.7% to 45.3%, which did not differ significantly by country. In community studies significant heterogeneity was reported in the seroprevalence between different age groups and the majority of studies reported there was no significant difference by gender.

**Conclusion:**

This review demonstrates a wide heterogeneity in reported seroprevalence of SARS-CoV-2 antibodies between populations. Continued evaluation of seroprevalence is required to understand the impact of public health measures and inform interventions including vaccination programmes.

## Introduction

On 11^th^ March 2020 the World Health Organization (WHO) declared the spread of the novel SARS-CoV-2 virus as a pandemic [1]. SARS-CoV-2 is thought to spread mainly by respiratory droplets, airborne transmission and some evidence also suggests spread via fomites [2–4]. SARS-CoV-2 causes varying degrees of illness from mild symptoms including fatigue and myalgia to acute respiratory failure and death [5]. As the pandemic unfolded evidence emerged that a large number of individuals are asymptomatic with SARS-CoV-2 infection [6, 7]. It has been suggested that asymptomatic transmission of SARS-CoV-2 could account for at least a third and up to 59% of transmission across the world [4].

In order to control the spread of SARS-CoV-2 it is important to understand the extent to which different populations have already been exposed to the virus, especially as a large number of infections are asymptomatic and therefore may not have been tested for SARS-CoV-2 at the time of infection. Many countries, organizational bodies and facilities have turned to mass testing to estimate the spread of infection and inform public health measures [8, 9]. One such testing strategy is by nasal and throat swabbing to detect viral RNA which has been piloted in England [10]. A further method is mass testing of the population for antibodies against SARS-CoV-2 [11]. Several tests for immunoglobulin G (IgG), immunoglobulin A(IgA) and immunoglobulin M(IgM) antibodies against SARS-CoV-2 have recently been developed. These broadly include enzyme linked immunosorbent assays, chemiluminescence immunoassays (CLIA) and point of care lateral flow assays [12].

Seroprevalence studies have been used in the past to help with outbreak responses. During a recent Ebola outbreak, seroprevalence studies were performed to gain further information on the immune response and immune protection [13]. Seroprevalence studies have also been used for infections such as rubella, mumps and measles to map resurgence and to gain further information on how public health strategies can target high risk populations [14]. Moreover, seroprevalence studies provide valuable information for vaccination strategies as they help to quantify how much of a population has been exposed to the virus helping to achieve herd immunity and helping to identify populations who may be at greater risk of infection.

By estimating the seroprevalence in different populations we can use the results to understand transmission dynamics, herd immunity and the immune response over time. These studies can help to guide the public health response to ultimately help prevent the spread of SARS-CoV-2. Here we present the results of a systematic review on the seroprevalence of SARS-CoV-2 in Europe.

## Methods

### Search strategy and selection criteria

This systematic review and meta-analysis adheres to the Preferred Reporting Items for Systematic Reviews and Meta-Analyses (PRISMA) guidelines [15]. The protocol was registered on the University of York database for Prospectively Registered Systematic Reviews (PROSPERO: 2020 CRD42020212149). We systematically searched electronic data sources from (01/12/2019) until (30/09/20) using search terms on seroprevalence and SARS-CoV-2. We searched the following electronic databases: Web of Science, MEDLINE, EMBASE, SCOPUS and Cochrane Database of Systematic Reviews. We also searched database search engine EBSCO to search the following databases: EBSCOhost e-book collection, biomedical reference collection, CINAHL plus and MEDLINE Complete. We conducted a secondary search by searching the reference lists of included studies for relevant articles.

Furthermore, we searched the grey literature. Firstly, we searched pre-print articles in the electronic database search engine EPMC to search pre-print databases including MedRXIV and BioRXIV. Secondly, we then used the database OpenGrey to search research reports, doctoral dissertations, conference papers and other forms of grey literature. Thirdly we searched the websites of national and international health agencies for reports relating to the seroprevalence of SARS-CoV-2 (World Health Organization, European Centres for Disease Control, Public Health England, Department of Health and Social Care in UK). Finally, we conducted a google search for further government reports.

Search terms were developed alongside a health science librarian (Table 1).

**Table 1:** Search strategy.

Search Strategy	
1	COVID-19 OR SARS-CoV-2
2	antibod* OR immun*
3	Seroprevalence
4	EUROPE
5	1 AND 2 AND 3 AND 4
6	animals NOT humans
7	4 NOT 5

Studies were included if they were written in English and published between 1/12/19–30/09/20 and were cross-sectional or cohort studies conducted in Europe. We defined European countries as those listed on the WHO regional office for Europe website [16]. Vaccine evaluations and randomised controlled trials were excluded.

### Data extraction

Titles and abstracts of retrieved studies were de-duplicated and screened independently by two reviewers to identify if they met the inclusion criteria. Screened references then underwent full text review by two reviewers independently. Any disagreement between them over the eligibility of studies was resolved through discussion with a third reviewer.

A standardised data extraction form was used. Data was extracted on the characteristics of the study (country, date, setting, selection method, funder), antibody assay employed, specificity and sensitivity of the assay, sample characteristics (age, gender, ethnicity, co-morbidities) and prevalence. Data extraction was carried out autonomously by the reviewers and consensus was sought between the team. Data was recorded on the type of funding the studies received; this included government funding, research grants with some studies receiving no external funding.

### Assessment of the methodological quality of included studies

We assessed the risk of bias using an adapted version of the Hoy et al modified Risk of Bias Tool, as used by Nguyen et al [17, 18]. This is a tool designed to assess the risk of bias in population-based prevalence studies. It uses a scoring system to assess the external and internal validity of the study. Studies that score 0–3 points are classified as low risk, 4–6 medium risk and 7–9 high risk. Two reviewers independently applied the criteria. Disagreements were resolved through discussion. After reviewing the relevant literature, we did not perform traditional asymmetry tests and funnel plots for assessing publication bias, as the meta-analysis we conducted was a summary of proportions and not a comparison of treatments/interventions [19, 20].

### Data analysis

We provide a narrative synthesis of the findings of all included studies, study population characteristics, antibody assays used and seroprevalence estimates for each study. A narrative synthesis aims to summarise the findings of multiple studies in the form of words and text [21]. Studies were categorized into subgroups including those that examined health care workers (HCWs), community studies, outbreaks, seroprevalence in children/schools and seroprevalence studies performed in pregnant women. We included studies in the subgroup outbreak if they investigated the seroprevalence following a sudden increase in SARS-CoV-2 cases related to time and place in a particular population [22]. We used Metaprop in STATA version 14, statistical software (Stata Corp. College Station, TX, USA), package to perform a random–effects meta-analysis of seroprevalence amongst health care workers (HCWs) stratified by country. The random effect model used the DerSimonian and Laird method with study heterogeneity estimated from the inverse-variance fixed-effect model. The pooled effect estimate was calculated after Freeman-Tukey Double Arcsine Transformation and Wilson score 95% confidence intervals were calculated for individual studies. We also performed a random-effects meta-analysis of seroprevalence amongst health care workers (HCWs) stratified by their risk of exposure to SARS-CoV-2 infected patients. HCWs were categorised as high risk if they worked with patients with known SARS-CoV-2 infection, medium risk if they had patient contact but without known SARS-CoV-2 infection and low risk if they had no patient contact (e.g., laboratory staff and administrative staff). If studies included participants from a mixture of risk groups they were categorised as medium risk. Heterogeneity was measured using the *I*^2^ statistic which describes the percentage of total variation due to inter-study heterogeneity. Tests of heterogeneity were undertaken within the sub-groups and for the overall meta-analysis. Sensitivity analysis was done by removing those studies with a moderate risk of bias score. This had no effect of the *I*^2^ value, so these studies were included in the final meta-analysis.

## Results

**Fig 1:**
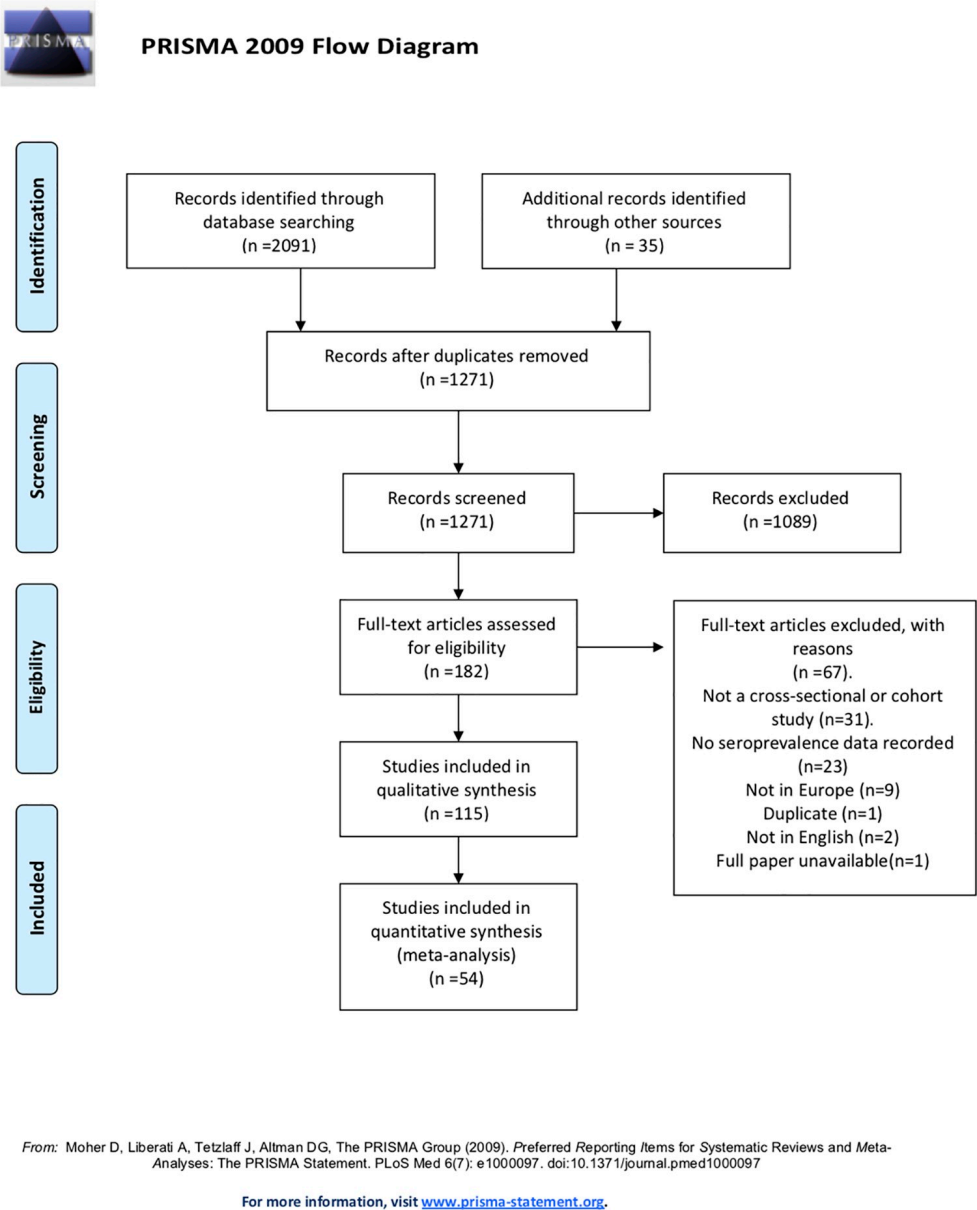
PRISMA flow chart.

The literature search yielded 2126 articles. After removing duplicates and excluding studies based on their abstract or through full text examination 115 studies were identified as eligible (Fig 1). The 115 articles spanned 17 countries in Europe and estimated the seroprevalence of SARS-CoV-2 from serum samples dated from November 2019 –August 2020. The 115 articles reviewed the seroprevalence in approximately 516,361 samples; among included studies 59.92% of subjects were females and the overall age range of participants was 0–90+ years (Table 2).

**Table 2:** Included studies, dates of sampling, population studied and overall seroprevalence.

Country	Author, year	Time period	Population	Total Seroprevalence (95% CI)
	Orth-Höller et al 2020[23]	20th-27th March	Primary and Secondary care physicians in Tyrol	5% (3.3–7.7)
	Knabl et al 2020[24]	April 21st- April 27th	Residents in Ischgl/Tyrol	42.4% (39.8–44.7)
Austria	Fuereder et al 2020 [25]	21st March - 4th June	HCWs and patients the Division of Oncology, Medical University of Vienna, Austria between 1 April and 4 June 2020.	HCWs—3.2% (0.4–11.2%) and patients 2.4% (0.3–8.3%)
	Reiter et al 2020[26]	Not recorded but published on medRxiv in July	Staff members of the Division of Nephrology and Dialysis, Department of Medicine III, at the Medical University of Vienna, Austria.	25.5% (20.4–31.5)
	Herzog et al 2020[27]	30th March -5th July	Residual sera from ten private diagnostic laboratories in Belgium.	30 March– 5 April 2.90% (2.3–3.4%) .20–26 April 6% (5.1–7.1%). 18–25 May 6.9% (5.9–8). 8–13 June 5.5% (4.7–6.5%). 29 June– 4 July 4.5%(3.7–5.4%)
	Berardis et al 2020[28]	16th April 16 and 19th May	Cystic Fibrosis (CF) patients followed in the CF reference centre of the Cliniques universitaires Saint-Luc (Brussels).	2.70%
Belgium	Steensels et al 2020[29]	22nd April - 30th April	HCWs at Hospital East-Limburg	6.40%
	Martin et al 2020[30]	15thApril-18th May	HCWs on COVID wards in Centre Hospitalier Universitaire Saint-Pierre (in Brussels	11–12%
	Desombere et al[31]	6th - 10th May	HCWs in Belgium	8.40%
	Blairon et al 2020[32]	25th May 25–19th June	HCWs at network of Iris hospitals South, Brussels, Belgium	14.60%
	Jerković et al 2020[33]	23rd - 28th April	Factory employees living in the Split-Dalmatia and Šibenik-Knin County	1.27% (0.77–1.98%)
Croatia	Vilibic-Cavlek et al 2020[34]	25th April - 24th May	HCWs and allied professions	2.7% based on IgG
	Iversen et al 2020 [35]	15th April and 23rd April	HCWs and blood donors in the Capital Region of Denmark	HCWs—4.04% (3·82–4·27). Blood Donors 3.04% (2.58–3.57)
	Erikstrup et al 2020[36]	6th April to 3rd May	Blood Donors in Denmark	2% (1.8–2.2)
	Egerup et al 2020 [37]	4^th^ April– 3^rd^ July	Women giving birth, their partners and newborns at University Hospital Hvidovre Obstetric and Gynaceology Unit	Women giving birth 2.85% (1.87%-4.25%) Partners 3.8% (2.6%-5.5% Newborns 1.4%
Denmark	Jespersen et al 2020[38]	May 18th until June 19th	HCWs and administrative staff that work in hospitals in the Central Denmark Region	3.7% (3.5%–4.0%)
	Laursen et al 2020[39]	22nd June to 10th August	Emergency and non-emergency HCWs employed by Falck in Sweden and Denmark	Denmark—2.8%. Sweden 8.3%
	Petersen et al 2020[40]	27th April– 1st May	Residents of the Faroe Islands	0.6% (0.2%–1.2%)
	Germain et al 2020[41]	1st November 2019 to 16th March 2020	All tissue donors at Lille Tissue bank	1.70%
	Solodky et al 2020[42]	1st March -16th April	HCWs and cancer patients	HCWs 5.4%. Cancer patients 5.9%
	Grzelak et al 2020[43]	20th March	A cohort of pauci-symptomatic individuals in Crepy-en-Valois. Blood donors from the Etablissement Français du Sang in Lille (France)	Blood donors mean of 3%. Pauci symptomatic cohort mean of 32%.
	Fontanet et al 2020 [44]	30^th^ March -4^th^ April	Students, teachers and non-teaching staff in a high school following an outbreak	25.86% (22.6%-29.4%)
	Carrat et al 2020[45]	1st April - 27th May	Residents from Ile-de-France or Grand Estor Nouvelle-Acquitaine	6.70%
France	Gallian et al 2020[46]	Last week of March to the first week of April	Blood Donors	2.70%
	Sermet et al 2020[47]	1st April - 1st June	Non COVID paediatric patients consulting or hospitalized in a paediatric tertiary health care department of the Assistance Publique-Hoôpitaux de Paris	22.19%
	Lisandru et al 2020[48]	16^th^ April– 15^th^ June	Residual serum samples in Corsica	5.27% (4.33%-6.35%)
	Fontanet et al 2020[49]	28th - 30th April	Pupils, their parents and relatives, and staff of primary schools exposed to SARS-CoV-2 in February and March 2020 in a city north of Paris, France	10.40%
	Mattern et al 2020[50]	4th May - 31st May	Patients admitted to the delivery room at Antoine Béclère Hospital maternity ward (Paris area, France)	8.00%
	Péré et al 2020[51]	2nd May - 26th June	HCWs at Hôpital Européen Georges Pompidou	12.20%
	Simon et al 2020[52]	February—April 2020	Patients on immunomodulatory imide drugs (IMIDs) with and without continuous cytokine blockade. HCWs and a cohort of healthy participants unrelated to health care	Healthy participants 2.27% HCWs 4.2%. IMIDs on cytokine inhibition 0.75%. IMIDs not on cytokine inhibition 3.08%
	Fischer et al 2020[53]	9th March - 3rd June	Blood donors in three German federal states North Rhine-Westphalia, Lower Saxony and Hesse	0.91% (0.58–1.24%)
	Brandstetter et al 2020[54]	20th March	HCWs at university children’s and maternity hospital in Regensburg	16.90%
	Brehm et al 2020[55]	20th March - 24th July	Employees of University Medical Center Hamburg-Eppendorf	1.8% (1–2.5%)
	Behrens et al 2020[56]	23rd March - 17th April	HCWs involved in COVID-19 patient care	0.90%
	Korth et al 2020[57]	25th March - 21st April	HCWs at University Hospital Essen	1.60%
	Streeck et al 2020[58]	31st March - 6th April	Residents of Gangelt	13.6% based on IgG
Germany	Krähling et al 2020[59]	6th April - 14th April	Employees of Infraserv Höchst, a large industrial site operator in Frankfurt am Main	0.50%
	Schmidt et al 2020[60]	20th April - 30th April	Employees BDH-Clinic Hessisch Oldendorf	2.86%
	Aziz et al 2020[61]	24th April -30th June	Individuals enrolled in the Rhineland Study an ongoing community- based prospective cohort study in people aged 30 years and above in the city of Bonn, Germany. Group I all living participants who had been enrolled in the Rhineland Study until March 18, 2020.Group II individuals who were eligible for but had not yet participated in the Rhineland Study.	Group I: 0.97% (0·72−1·30). Group II: 1.94% (0·84−4·42)
	Weis et al 2020[62]	12th May - 22nd May	Residents in Neustadtam Rennsteig	8.40%
	Bahrs et al 2020[63]	19 th May– 20^th^ May	HCWs in a University Hospital	2.7% (1.6% -4.3%)
	Armann et al 2020[64]	25th May - 30th June	Students grade 8–11 and their teachers in 13 secondary schools in eastern Saxony	0.60%
	Epstude et al 2020[65]	15th June - 30th June	HCWs and housekeeping staff in an oncology unit	3.10%
	Hoffmann et al 2020[66]	20th July	HCWs in a standard care hospital in Oberspreewald-Lausitz	1.30%
	Bogogiannidou et al 2020[67]	March and April	Individuals who visited the laboratories for a check-up, chronic disease follow-up or other reasons unrelated to COVID-19 in the whole of Greece	March: 0.24% (0.03–0.45%). April 0.42% (0.23–0.61%)
Greece	Psichogiou et al 2020[68]	30th April - 15th May	HCWs across two hospitals in Greece	1.00%
	Tsitsilonis et al 2020[69]	June-July 2020	Student and staff at National and Kapodistrian University of Athens	1.00%
Iceland	Gudbjartsson et al 2020[70]	January—July	Two groups of qPCR-positive Icelanders and six groups of Icelanders who had not been qPCR-tested or who had been tested and had received a negative result	4.22%
	Plebani et al 2020[71]	22nd February - 29th May	HCWs in several Structures of the National Healthcare Service of the Veneto Region	4.6% (4.1–5.0%)
	Valenti et al 2020[72]	24th February - 8th April	Blood donors in Milan	5.07%
	Pancrazzi et al 2020[73]	17th - 21st March	Patients in the Emergency Room and from subjects undergoing health surveillance by territorial and hospital prevention departments in Tuscany	13.00%
	Vena et al 2020 [74]	March and April	Residents living in Liguria and Lombardi regions	11.00%
	Norsa et al 2020[75]	March- July	Patients with IBD on biologic treatment	21.10%
	Percivalle et al 2020[76]	18th March - 6th April	Registered blood donors in Lodi Italy	23.00%
	Lahner et al 2020[77]	18th March–27th April	HCWs in a teaching hospital in Rome	0.70%
	Fusco et al 2020[78]	23rd March - 2nd April	HCWs working in a specialist infectious disease setting, the ‘D. Cotugno’ hospital in Naples, Italy.	1.70%
	Sotgiu et al 2020[79]	2nd - 16th April	HCWs at San Paulo University General Hospital	7.4% based on IgG
	Amendola et al 2020[80]	15th April	HCWs in Buzzi Hospital	5.13%
Italy	Carozzi et al 2020 [81]	20th April	HCWs across 6 health facilities in Tuscany	2.4% only including positive results and not borderline results
	Comar et al 2020[82]	Published in medRxiv in April	All employees of the Mother and Child Research Hospital Burlo Garofolo	7.2% just including positive and not borderline results.
	Cosma et al 2020[83]	16th April - 4th June	Pregnant women attending for first trimester screening (11‐13 weeks of gestation) at Sant’Anna Hospital, Turin, Piedmont	10.10%
	Sandri et al 2020[84]	28th April - 16th May	Employees of 7 different hospitals, located across the Lombardy region	11.21%
	Fiore et al 2020[85]	1st May - 31st May	Blood donors in Apulia region, South Eastern Italy	0.99%
	Pagani et al 2020[86]	18th May - 7th June	Residents of Castiglione D’Adda	22.6% (17.2–29.1%)
	Paderno et al 2020[87]	No date recorded	All staff working in a COVID-19-free Otolaryngology Department in Italy	6.90%
	Tosato et al 2020[88]	Not recorded but published on medRxiv in May	HCWs working in the Department of Laboratory Medicine	4.50%
Netherlands	Westerhuis et al 2020[89]	2nd March - 3rd April	Patients of the Erasmus Medical Centre in Rotterdam	March: 0.7%. April: 3%
	Slot et al 2020[90]	1st - 15th April	Plasma donations	2.60%
Luxembourg	Snoeck et al 2020[91]	15th April - 5th May	Population of Luxembourg	1.97%
Portugal	Figueiredo-Campos et al 2020[92]	6th April - 10th July	Hospitalised patients and HCWs who tested positive for SARS-CoV-2 by PCR, healthy post-COVID-19 volunteers and staff of the University of Lisbon	Patients: 51%, HCWs 100%, plasma donations 88%, University staff 1.5%
	Dacosta-Urbieta et al 2020[93]	March—April	HCWs of the Paediatric Department of the Hospital Clínico Universitario de Santiago de Compostela	4.00%
	Garcia-Basteiro et al 2020[94]	28th March - 9th April	HCWs at Hospital Clínic of Barcelona	9.3% (7.1–12%)
	Valdivia et al 2020[95]	13th - 30th April	HCWs at Hospital Clínico Universitario of Valencia	3.50%
	Galán et al 2020[96]	14th -27th April	All HCWs Hospital Universitario Fundación Alcorcón	31.60%
	Crovetto et al 2020[97]	14th April-5th May	Pregnant women consecutively attending first trimester screening or delivery	14.30%
	Garralda Fernandez et al 2020[98]	14th April - 13th May	HCWs at Hospital Universitario de Fuenlabrada	16.9% based on IgG
Spain	Olalla et al 2020[99]	15th - 25th April	HCWs of the Costa del Sol Hospital in Marbella of the units involved in patient care with COVID-19	2.20%
	Martín et al 2020[100]	20th April	General practitioners (GP) and primary care nurses in the Healthcare Area of León, who worked in health centres or nursing homes.	5.90%
	Montenegro et al 2020[101]	21 April to 24 April 2020 (Study population A) and from 29 April to 5 May 2020 (Study population B)	Population A: individuals registered in a primary health care centre, from a community area of Barcelona, Spain. Population B: Patients from GPs in Barcelona presenting with mild-moderate symptoms of COVID but no diagnosis of COVID	Population A: 5.47% (3.44–8.58%). Population B: 38.49% (34.78%-42.33%)
	Moncunill et al 2020[102]	April—May	HCWs from Hospital Clínic de Barcelona	14.50%
	Soriano et al 2020[103]	April—May	Asymptomatic adults in Madrid	13.80%
	Pollan et al 2020[104]	27th April - 11th May	Spanish households	5% (4.7–5.4%) based on POC testing
	Martínez et al 2020[105]	15^th^ April– 15^TH^ June	Essential workers in Madrid who were known to have exposure to SARS-CoV-2 contacts or had reported COVID-19 compatible symptoms	33.69% (29.27%– 38.21%)
	Barallat et al 2020[106]	4th - 22nd May	HCWs of the Catalan Institute of Health (ICS) Northern Metropolitan Area of Barcelona	10.30%
	Cabezón-Gutiérrez et al 2020[107]	1st- 19th June	Oncology outpatients who attended the medical oncology consultation of the University Hospital of Torrejón	31.40%
	Castro Dopico et al 2020[108]	30th March - 23rd August	Blood Donors and pregnant women in Stockholm	13.7% (9.5–19.3%) estimated by ENS Learner
	Rudberg et al 2020[109]	24th April - 8th May	HCWs at Danderyd Hospital	19.10%
	Lindahl et al 2020[110]	20th April	Employees of elderly care homes situated in Stockholm city and its suburbs	23% (20.4–25.7%)
Sweden	Roxhed et al 2020[111]	20th April	Households in Stockholm	4.4% (2.4%-6.3%) for IgG
	Lidström et al 2020[112]	27^th^ May– 25^TH^ June	All healthcare staff including support staff, in the Region Uppsala	6.60%
	Lundkvist et al 2020[113]	17th-18th June	Residents of Norra Djurgårdsstaden and Tensta in Stockholm	15.00%
	Laursen et al 2020[39]	22nd June to 10th August	Emergency and nonemergency HCWs employed by Falck in Sweden and Denmark	Denmark—2.8%. Sweden 8.3%
	Emmenegger et al 2020[114]	February—July	Patients entering the University Hospital of Zurich and healthy blood donors in Zurich	Hospitalised patients: March 2020 0.3% (0.1% - 0.5%). April 2020 1.4% (1.0%-1.7%). Blood donors: April 1.2% (0.7%-1.8%). May 1.6% (1.0%-2.3%) estimated by quadratic discriminant analysis
Switzerland	Stringhini et al 2020[115]	6th April - 9th May	Residents from the Canton of Geneva	7.90%
	Ulyte et al 2020[116]	June-July	Schools in the Canton of Zurich	2.8% (1.4–6.1%)
Turkey	Alkurt et al 2020[117]	30th May - 6th June	HCWs in the University of Health Sciences Umraniye Teaching and Research Hospital (UEAH), Istanbul University-Cerrahpasa, Cerrahpasa Medical Faculty Hospital (Cerrahpasa), Darica Farabi Teaching and Research Hospital (Farabi)	12.30%
	Thompson et al 2020[118]	17th March - 19th May 2020	Blood donors in Scotland	3.17%
	Houlihan et al 2020[119]	26th March - 8th April	HCWs at University College London Hospitals (UCLH) who work in accident and emergency (A&E), COVID ward, acute medical unit (AMU), intensive care (ICU) or haematology	45.30%
	Pallett et al 2020[120]	8th April - 12th June	HCWs in two hospitals in London	39.30%
	Waterfield et al 2020[121]	16th April - 3rd July	Children of healthcare workers, aged between 2 and 15 years	6.90%
	Eyre et al 2020[122]	23rd April - 8th June	Hospital staff at Oxford University Hospitals NHS Foundation Trust	10.70%
	Shields et al 2020[123]	24th -25th April	HCWs at University of Birmingham and University Hospitals Birmingham NHS Foundation Trust	24.40%
UK	Clarke et al 2020[124]	27th April - 7th May	Patients receiving in centre haemodialysis (ICHD) within two units affiliated with Imperial College Renal and Transplant Centre	36.20%
	Wells et al 2020[125]	27th April - 2nd June	Participants of the Twins UK Cohort Study	12.00%
	The Government of Jersey 2020[126]	29th April - 5th May	Residents in Jersey over 16 years of age	3.1% (+/- 1.3%)
	Poulikakos et al 2020[127]	4th-6th May	HCWs from renal and biochemistry department in a tertiary hospital in the northwest of England	6.00%
	Khalil et al 2020[128]	15th - 28th May	HCWs at Portland Hospital for Women and Children	22.00%
	Grant et al 2020[129]	15th May - 5th June	HCWs at Whittington Health	31.64%
	Ladhani et al 2020[130]	20th May	Residents and staff of the six care homes following an outbreak of COVID-19	77.9% (73.6–81.7%)
	Biobank 2020[131]	27th May - 14th August	UK Biobank participants	8.2% (7.9%-8.7%)
	Public Health England 2020[132]	May	Healthy adult blood donors, supplied by the NHS Blood and Transplant (NHS BT)	8.5% (6.9–10%) this is adjusted for the sensitivity and specificity of the Euroimmune assay
	Mulchandani et al 2020 [133]	1st June - 26th June	Police and Fire and Rescue services, healthcare workers and healthcare workers with previously positive for SARS-CoV-2	Police and Fire and Rescue services: 10.6%. HCWs: 23.3%
	Nsn et al 2020[134]	20th June	Residents in 4 nursing homes in UK where a covid-19 outbreak happened	71.80%
	Favara et al 2020[135]	June—July	HCWs with direct patient contact working in an oncology unit in either of the following hospitals the Queen Elizabeth Hospital Kings Lynn NHS Foundation Trust (QEH), North West Anglia NHS Foundation Trust (Peterborough City Hospital, NWA), and Cambridge University Hospitals NHS Foundation Trust (CUH).	13.3% using Luminex assay on day 28
	Ladhani et al 2020[136]	June—July	Teachers and students in 131 schools across England	11.7% (10.5–13.3%)
	Ward et al 2020 [137]	20th June - 13th July	Residents in England over the age of 18 years	6% (5.8–6.1%) adjusted for test performance and re-weighted for sampling

Abbreviations: A&E–accident and emergency; AMU–acute medical unit

CF—cystic fibrosis; HCWs–health care workers; IBD—inflammatory bowel disease; ICHD–in centre haemodialysis; IMIDs - immunomodulatory imide drugs; ITU—intensive care unit; NHS–national health service; qPCR–quantitative polymerase chain reaction; UK–United Kingdom.

### Assessment of the methodological quality of included studies

Each study underwent a risk of bias assessment using the modified Hoy et al risk of bias tool [18]. Twenty nine of the 115 studies were deemed to be at moderate risk of bias [31, 34, 42, 44, 47, 54, 56, 57, 67, 69, 73, 74, 88, 95, 97, 100, 107, 108, 111, 116, 118, 121, 122, 125, 127, 128, 132, 134, 136]. This was often due to lack of information about the sampling frame, selection process and non-response bias. No studies scored high risk (Table 3).

**Table 3:** Assessment of the methodological quality of included studies.

Country	Author, year	Overall Risk of Study: high, moderate or low	Reason if moderate/high risk
	Orth-Höller et al 2020[23]	Low risk	
	Knabl et al 2020[24]	Low risk	
Austria	Fuereder et al 2020 [25]	Low risk	
	Reiter et al 2020[26]	Low risk	
	Herzog et al 2020[27]	Low risk	
	Berardis et al 2020[28]	Low risk	
Belgium	Steensels et al 2020[29]	Low risk	
	Martin et al 2020[30]	Low risk	
	Desombere et al [31]	Moderate risk	Not enough information given about the sampling frame, definition of a positive result, selection process, type of test used, if the same test was used on all participants and non-response bias.
	Blairon et al 2020[32]	Low risk	
	Jerković et al 2020[33]	Low risk	
Croatia	Vilibic-Cavlek et al 2020[34]	Moderate risk	Not enough information given about the sampling frame, selection process and non-response bias.
	Iversen et al 2020 [35]	Low risk	
	Erikstrup et al 2020[36]	Low risk	
	Egerup et al 2020 [37]	Low risk	
Denmark	Jespersen et al 2020[38]	Low risk	
	Laursen et al 2020[39]	Low risk	
	Petersen et al 2020[40]	Low risk	
	Germain et al 2020[41]	Low risk	
	Solodky et al 2020[42]	Moderate risk	Not enough information given about the sampling frame, selection process and non-response bias.
	Grzelak et al 2020[43]	Low risk	
	Fontanet et al 2020 [44]	Moderate risk	Not enough information on case definition, sampling frame and non -response bias
	Carrat et al 2020[45]	Low risk	
France	Gallian et al 2020[46]	Low risk	
	Sermet et al 2020[47]	Moderate risk	Not enough information given about the sampling frame, selection process and non-response bias.
	Lisandr et al 2020 [48]	Low risk	
	Fontanet et al 2020[49]	Low risk	
	Mattern et al 2020[50]	Low risk	
	Péré et al 2020[51]	Low risk	
	Simon et al 2020[52]	Low risk	
	Fischer et al 2020[53]	Low risk	
	Brandstetter et al 2020[54]	Moderate risk	Not enough information given about the sampling frame, selection process and non-response bias.
	Brehm et al 2020[55]	Low risk	
	Behrens et al 2020[56]	Moderate risk	Not enough information given about the sampling frame, selection process and non-response bias.
	Korth et al 2020[57]	Moderate risk	Not enough information given about the sampling frame, selection process and non-response bias.
	Streeck et al 2020[58]	Low risk	
Germany	Krähling et al 2020[59]	Low risk	
	Schmidt et al 2020[60]	Low risk	
	Aziz et al 2020[61]	Low risk	
	Weis et al 2020[62]	Low risk	
	Bahrs et al 2020 [63]	Low risk	
	Armann et al 2020[64]	Low risk	
	Epstude et al 2020[65]	Low risk	
	Hoffmann et al 2020[66]	Low risk	
	Bogogiannidou et al 2020[67]	Moderate risk	Not enough information given about definition of a positive result, non-responder bias and discrepancies in the tables.
Greece	Psichogiou et al 2020[68]	Low risk	
	Tsitsilonis et al 2020[69]	Moderate risk	Not enough information given about the sampling frame, selection process and non-response bias.
Iceland	Gudbjartsson et al 2020[70]	Low risk	
	Plebani et al 2020[71]	Low risk	
	Valenti et al 2020[72]	Low risk	
	Pancrazzi et al 2020[73]	Moderate risk	Not enough information given about the sampling frame, selection process and non-response bias.
	Vena et al 2020 [74]	Moderate risk	Not enough information given about the sampling frame, selection process and non-response bias.
	Norsa et al 2020[75]	Low risk	
	Percivalle et al 2020[76]	Low risk	
	Lahner et al 2020[77]	Low risk	
	Fusco et al 2020[78]	Low risk	
	Sotgiu et al 2020[79]	Low risk	
	Amendola et al 2020[80]	Low risk	
Italy	Carozzi et al 2020 [81]	Low risk	
	Comar et al 2020[82]	Low risk	
	Cosma et al 2020[83]	Low risk	
	Sandri et al 2020[84]	Low risk	
	Fiore et al 2020[85]	Low risk	
	Pagani et al 2020[86]	Low risk	
	Paderno et al 2020[87]	Low risk	
	Tosato et al 2020[88]	Moderate risk	Not enough information given about the sampling frame, selection process and non-response bias.
Netherlands	Westerhuis et al 2020[89]	Low risk	
	Slot et al 2020[90]	Low risk	
Luxembourg	Snoeck et al 2020[91]	Low risk	
Portugal	Figueiredo-Campos et al 2020[92]	Low risk	
	Dacosta-Urbieta et al 2020[93]	Low risk	
	Garcia-Basteiro et al 2020[94]	Low risk	
	Valdivia et al 2020[95]	Moderate risk	Not enough information given about the sampling frame, selection process and non-response bias.
	Galán et al 2020[96]	Low risk	
	Crovetto et al 2020[97]	Moderate risk	Not enough information given about the sampling frame, selection process and non-response bias.
	Garralda Fernandez et al 2020[98]	Low risk	
Spain	Olalla et al 2020[99]	Low risk	
	Martín et al 2020[100]	Moderate risk	High non-response rate and not enough information given about the sampling frame and selection process.
	Montenegro et al 2020[101]	Low risk	
	Moncunill et al 2020[102]	Low risk	
	Soriano et al 2020[103]	Low risk	
	Pollan et al 2020[104]	Low risk	
	Martínez et al 2020[105]	Low risk	
	Barallat et al 2020[106]	Low risk	
	Cabezón-Gutiérrez et al 2020[107]	Moderate risk	Not enough information given about the sampling frame, definition of a positive result, selection process and non-response bias.
	Castro Dopico et al 2020[108]	Moderate risk	Not enough information given about the sampling frame, definition of a positive result, selection process and non-response bias.
	Rudberg et al 2020[109]	Low risk	
	Lindahl et al 2020[110]	Low risk	
Sweden	Roxhed et al 2020[111]	Moderate risk	Not enough information given about the sampling frame, definition of a positive result, selection process and non-response bias.
	Lidstrom et al 2020 [112]	Low risk	
	Lundkvist et al 2020[113]	Low risk	
	Laursen et al 2020[39]	Low risk	
	Emmenegger et al 2020[114]	Low risk	
Switzerland	Stringhini et al 2020[115]	Low risk	
	Ulyte et al 2020[116]	Moderate risk	Not enough information given about the sampling frame, definition of a positive result, selection process and non-response bias.
Turkey	Alkurt et al 2020[117]	Low risk	
	Thompson et al 2020[118]	Moderate risk	Not enough information given about the sampling frame, definition of a positive result, selection process and non-response bias.
	Houlihan et al 2020[119]	Low risk	
	Pallett et al 2020[120]	Low risk	
	Waterfield et al 2020[121]	Moderate risk	Not enough information given about the sampling frame, definition of a positive result, selection process and non-response bias.
	Eyre et al 2020[122]	Moderate risk	High non-response rate and not enough information given if the same test was used on all participants and definition of a positive result.
	Shields et al 2020[123]	Low risk	
UK	Clarke et al 2020[124]	Low risk	
	Wells et al 2020[125]	Moderate risk	High non-response rate and not enough information given about the sampling frame and selection process.
	The Government of Jersey 2020[126]	Low risk	
	Poulikakos et al 2020[127]	Moderate risk	Not enough information given about the sampling frame, selection process and non-response bias.
	Khalil et al 2020[128]	Moderate risk	Not enough information given about the sampling frame, selection process and non-response bias.
	Grant et al 2020[129]	Low risk	
	Ladhani et al 2020[130]	Low risk	
	Biobank 2020[131]	Low risk	
	Public Health England 2020[132]	Moderate risk	Not enough information given about the sampling frame, selection process and non-response bias.
	Mulchandani et al 2020 [133]	Low risk	
	Nsn et al 2020[134]	Moderate risk	Not enough information given about the selection process and non-response bias.
	Favara et al 2020[135]	Low risk	
	Ladhani et al 2020[136]	Low risk	
	Ward et al 2020 [137]	Low risk	

### Results by population subgroups

#### Health care workers

54 studies included seroprevalence data among HCWs. These studies included data from 13 countries in Europe and were conducted between February 2020 and August 2020.

**Fig 2:**
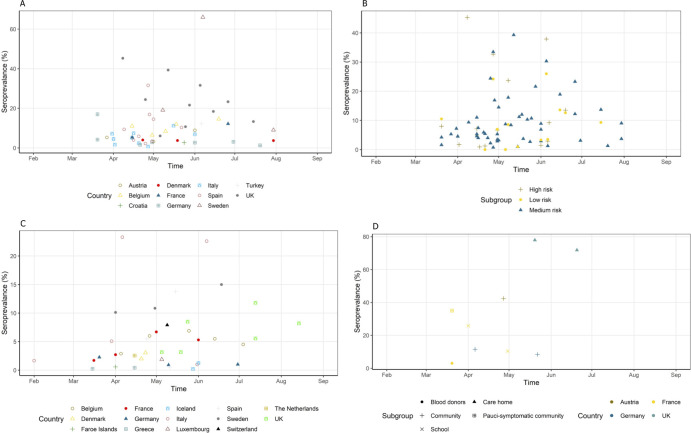
Seroprevalence of subgroups over time. (A): the seroprevalence of HCWs by country over time. (B): the seroprevalence of HCWs over time stratified by risk group. (C): the seroprevalence of community studies over time by country. (D): the seroprevalence of outbreak studies over time stratified by country and subgroup.

The lowest seroprevalence was seen in a teaching hospital in Rome, Italy during the months of March–April 2020, reporting a seroprevalence of 0.7% based on IgG antibodies and 0% based on IgM [77]. The highest seroprevalence (45.3%) was reported in March–April 2020 in a University Hospital in London [119]. Fig 2A shows the seroprevalence of HCWs by country over time. The majority of studies report a seroprevalence < 10% between March–August 2020. A few studies predominately based in the UK report a seroprevalence 20–45% among HCWs during this time period [26, 96, 119, 120, 123, 128, 129, 133].

Fig 2B shows the seroprevalence of HCWs categorised by their risk of exposure to SARS-CoV-2 patients over time. All subgroups with a seroprevalence of >30% belonged to either medium or high risk. The majority (67/79) of the subgroups had a seroprevalence of less than 20% regardless of their risk.

**Fig 3:**
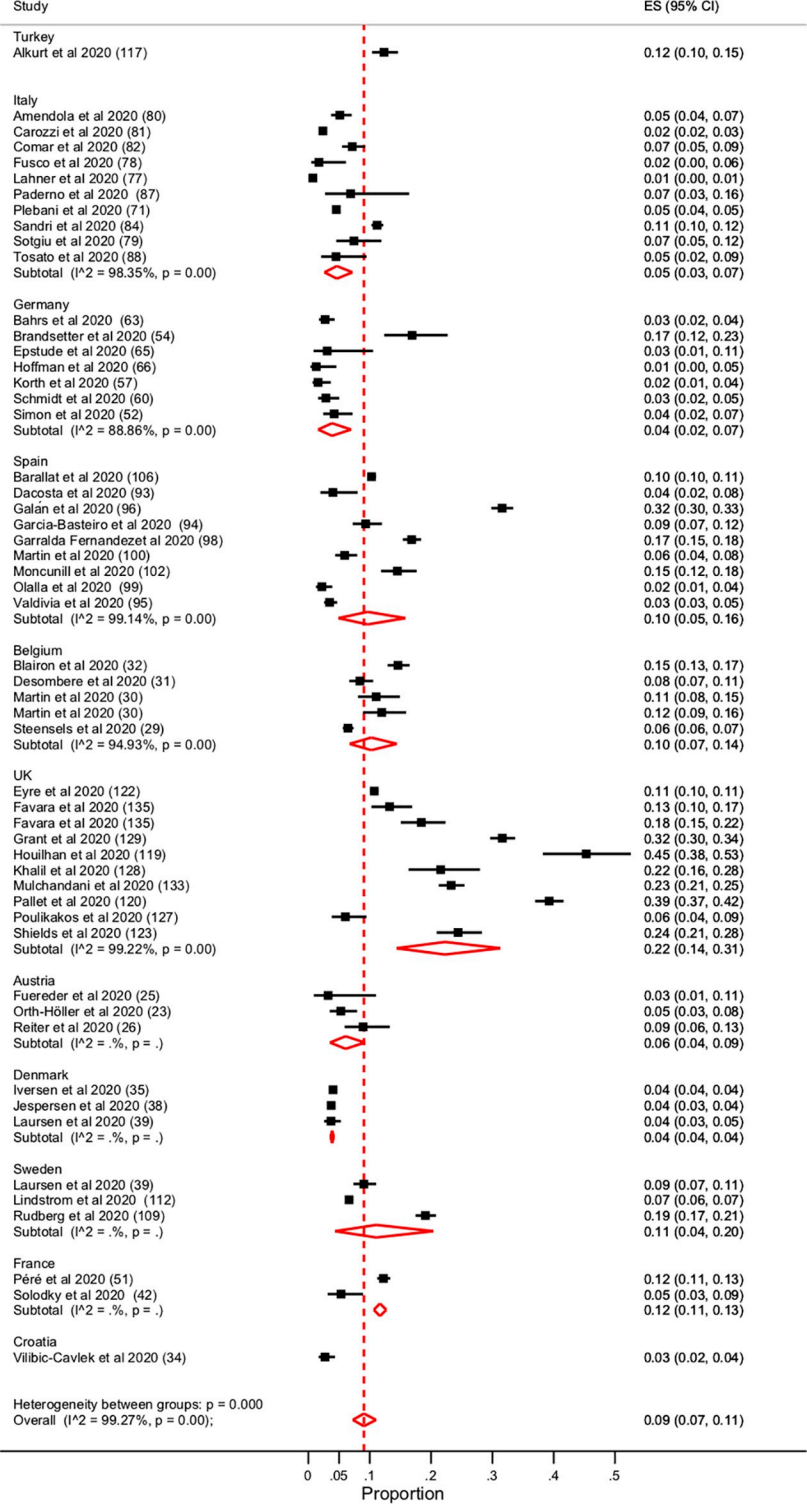
Forest plot of the seroprevalence among HCWs stratified by country.

There was no significant difference in seroprevalence amongst HCWs when stratified by country (Fig 3). There is a large amount of heterogeneity between the studies (I^2^ value = 99.27%, p = 0.00). There was no reduction in heterogeneity when moderate risk of bias studies were removed. Similarly, when the seroprevalence amongst HCWs was stratified by their risk of exposure to SARS-CoV-2 patients there was no significant difference (S1 Fig).

#### Community studies

In total 34 studies were set in the community, spanning 13 countries. The studies collected data between February 2020—August 2020. Ten of these studies collected samples from blood donors; five studies used residual serum samples from clinics, laboratories and hospital facilities; one study used tissues samples and the remaining studies were randomised population-based studies.

The overall lowest seroprevalence was reported in Greece in March 2020 of 0.24% (0.03–0.45%) [67]. The same study reported an increase in seroprevalence in April of 0.42% (0.23–0.61%). The overall highest seroprevalence was reported in Lodi, Italy during the months of March and April at 23% [76]. The majority of studies reported an overall seroprevalence of less than 10% during the months of February–August 2020 (Fig 2C).

Age. Many of the community studies report the seroprevalence among different age groups. There is significant heterogeneity between the results. In general, lower seroprevalences were reported at the extremes of age. Several studies report a higher seroprevalence among the over 50 age group [40, 52, 74, 85, 86, 104]. In contrast some studies report a higher seroprevalence in the less than 30 years age group, these include studies from Switzerland, the Netherlands, Denmark, France, Luxembourg and the UK [45, 90, 91, 115, 131, 137].

Gender. In the majority of community studies there was no significant difference identified by gender. However, two studies reported a significantly higher number of female participants having antibodies against SARS-CoV-2 [45, 74]. Carrat et al investigated the seroprevalence in three administrative regions of France Ile-de-France (IDF), Grand Est (GE) and Nouvelle-Aquitaine (NA), and reported a significant association of antibodies associated with the female gender only in Nouvelle-Aquitaine [45]. Similarly, Vena et al report a significantly higher seroprevalence among female participants in five administrative regions in Italy [74].

Blood donors. Many studies report the seroprevalence in blood donors as they are usually healthy individuals who represent the general population. There was a large variation in seroprevalence among blood donors between countries and over time.

The lowest seroprevalence in blood donors was reported in Germany between March and June 2020 of 0.91% (0.58–1.24%) [53]. In contrast Percivalle et al reported the highest seroprevalence amongst Italian blood donors in April, living in the Lodi Red Zone of 23.3% [76]. The Lombardi Red Zone is an area of 10 municipalities that were put in total social and commercial lockdown from the 23^rd^ February 2020 [76].The same study reports a seroprevalence of 1.67% in February 2020. In addition a study in the South East of Italy reports a seroprevalence on 0.99% in May 2020 [85].

Similar variations of estimates of seroprevalence were reported in blood donors in the UK. A study conducted in Scotland reported a seroprevalence of 3.17% between the months of March–May 2020 [118]. A seroprevalence of 8.5% (6.9–10%) was reported in blood donors across England in May [132].

Children/ Schools. Seven studies investigated the seroprevalence in school/university settings or among children only, across 5 different countries [49, 64, 69, 116, 121, 136]. Four of the studies examined the seroprevalence in schools [49, 64, 116, 136]. The lowest seroprevalence was reported in Germany; 0.6% among students in grade 8–11 and their teachers in 13 secondary schools in eastern Saxony between the months of May–June 2020 [64]. The highest seroprevalence of 11.7% was reported in students and teachers across schools in England between June-July 2020 (10.5–13.3%) [136].

Fontanet et al investigate the seroprevalence in a high school following an outbreak [44]. They report an overall seroprevalence of 25.86% among pupils, staff and parents of high school pupils [44]. Following the original outbreak Fontanet et al then investigated the seroprevalence among pupils and teachers in primary schools in the local area and reported an overall seroprevalence of 10.4% [49]. They noted that 41.4% of infected children had asymptomatic infection compared to 9.9% of seropositive adults [49].

One study examined the seroprevalence among university students and staff in Greece [69]. They reported an overall seroprevalence of 1%, with no significant difference by age, gender, school or position [69].

*Outbreaks*. Eight studies across four countries investigated the seroprevalence following an outbreak of SARS-CoV2 [24, 43, 44, 49, 58, 62, 130, 134]. Two of the studies were conducted in the UK and reported a high prevalence of antibodies against SARS-CoV-2 in residents and staff in care homes/nursing homes where there had been a recent SARS-CoV-2 outbreak [130, 134]. They report a high prevalence of antibodies against SARS-CoV-2. Ladhani et al estimated a seroprevalence of 77.9% (73.6–81.7%) and Nsn et al report a seroprevalence of 71.8% [130, 134].

Four studies report seroprevalences following outbreaks among blood donors or communities [24, 43, 58, 62]. They report much lower rates of seroprevalence compared to nursing home outbreaks. Grzelak et al investigated the seroprevalence of pauci-symptomatic individuals in Crepy-en-Valois France and blood donors in the surrounding region following an outbreak; they reported a seroprevalence of 3% in blood donors and 32% in the pauci-symptomatic individuals [43]. Similarly, studies in Germany following community outbreaks report low rates of seroprevalence among residents. Streeck et al reported a prevalence of SARS-CoV-2 antibodies of 13.6% and Weis et al reported a seroprevalence of 8.4% [58, 62]. Fig 2D shows the seroprevalence of these outbreak studies over time.

#### Pregnancy

Four studies examined the seroprevalence of SARS-CoV-2 in pregnant women [37, 50, 83, 97]. Two of these studies conducted in Italy between April–June 2020 reported a prevalence of SARS-CoV-2 antibodies of 10.1% and 14.3% in pregnant women in their first trimester screening or at delivery [83, 97]. Mattern et al estimated a seroprevalence of 8% among pregnant women admitted to the delivery room in France in May 2020 [50]. Mattern et al found that the seroprevalence among pregnant women was similar to that of the general public [50].

Egerup et al investigate the seroprevalence among pregnant women, their partners and newborn babies [37]. They report a seroprevalence of 2.85% among parturient women, 3.8% among partners and 1.4% among newborn babies [37]. They report no association between COVID-19 and obstetric or neonatal complications [37].

#### Assays

In total 47 different commercial assays and 22 in-house assays were used. The majority of studies used more than one assay. Of the commercial assays 11 were enzyme-linked immunosorbent assay (ELISA), seven were chemiluminescent microparticle immunoassays, two were based on flow cytometry and 27 were point of care tests (POC). The most used commercial assay was the SARS-CoV-2 (IgA/IgG) ELISA EUROIMMUN Medizinische Labordiagnostik, Lübeck, Germany (Table in S2 Table).

## Discussion

Our systematic review demonstrates a large variation in the seroprevalence of SARS-CoV-2 antibodies throughout Europe in the first half of 2020.

HCWs in the UK had a much higher seroprevalence compared to HCWs in the rest of Europe during the months of March and August 2020. There are nine studies which took place in UK and six of them reported a seroprevalence of more 20% among HCWs [119, 120, 123, 128, 129, 133]. In contrast, Italy reports a low seroprevalence among HCWs. Of 10 studies among HCWs in Italy, nine reported a seroprevalence of SARS-CoV-2 antibodies of less than 10% [71, 77–82, 87, 88]. Both countries included studies from a mixture of high, medium and low risk HCWs and during this time both countries had high numbers of SARS-CoV-2 infections.

In health care settings, the risk of HCWs of SARS CoV-2 exposure was determined by the COVID-19 caseload coming though the facility and the application of infection control measures. Infection control practices in relation to personal protective equipment (PPE) may in part explain some of the differences.

Between European countries there are differences in the recommended PPE. The UK government guidelines on PPE include the use of eye/face protection, filtering facepiece class 3 (FFP3) respirator, disposable fluid-repellent coverall, and disposable gloves for aerosol-generating procedures and higher-risk acute care areas. For all inpatient ward settings eye/face protection, fluid-resistant (type IIR) surgical mask (FRSM), disposable plastic apron and disposable gloves are recommended [138].

In comparison, the National Institute of Health in Italy recommended that all HCWs wear a full-length gown with long sleeves, hairnet, goggles, gloves and surgical mask in the case of low-risk patients, and hairnet, googles or face-shield, FFP3 mask, water-resistant gown with long sleeves, and two pairs of gloves (second one covering the wrist of gown sleeves) for high risk patients and SARS-CoV-2 positive patients [77].

The main difference in the PPE provided in both countries was that Italy recommended that for all HCWs a full-length gown should be worn, a hairnet and an FFP3 mask when caring for all high-risk patients and all SARS-CoV-2 positive patients. As evidence now supports aerosol spread of SARS-CoV-2, reviews have been conducted investigating the protectiveness of surgical face masks and FFP3 masks. One such study concluded that surgical face masks provide no protection against aerosol particles, in comparison FFP3 masks provided adequate protection [139].

Although the availability and differences in PPE across European countries may partly explain the difference in seroprevalence seen in HCWs, there are other factors that require consideration. For example, differences in public health strategies and the time of their implementation such as the public wearing face masks, closure of educational settings and other public facilities. Furthermore, differences in adherence to infection control measures such as hand hygiene and social distancing could also explain the difference in seroprevalence seen among HCWs across Europe.

Our systematic review found that in the majority of studies in Europe there was no difference in seroprevalence between female and male participants. Our findings are in keeping with a meta-analysis which showed there was no difference in the proportion of males and females with confirmed COVID-19 [140].

Throughout the current pandemic there has been debate on the role of children in the transmission of SARS-CoV-2 and the need for school closure to slow the pandemic. In this review three studies were conducted in schools not involved in an outbreak of SARS-CoV-2. A study in Germany reported the lowest seroprevalence among students and teachers in a school of 0.6% considered by the authors to be in keeping with local surveillance data of the surrounding community [64]. Ulyte et al reported that seroprevalence is inversely related to age in their school study [116]. They conclude this could be due to the lack of social distancing among young children and differences in immune response [116]. In contrast Ladhani et al reported no significant difference between the seroprevalence in students compared to staff [136]. However all studies concluded that there was no major transmission in schools and that the majority of children were asymptomatic or had mild symptoms [64, 116, 136].

However, since these studies have been conducted there has been the introduction of the SARS-CoV-2 variants; B 1.1.7 and B 1.617.2. An increase in these variants have been seen among children and young adolescents with outbreaks occurring in schools and child-care facilities [141, 142].

As new variants emerge it would be reasonable for more school-based studies investigating the seroprevalence among staff and students to be conducted to fully understand transmission dynamics and immune response over time. Viewed within the context of local community data this will help to inform public health strategies to both protect children and to minimise transmission in the wider community.

In studies conducted during local outbreaks, there was a noticeable difference between those conducted in care/nursing homes compared to community and school settings. Those that took place in care/ nursing homes reported a seroprevalence as high as 77.9%, whereas those in a community setting reported a seroprevalence ranging from 3% - 42.4% and those in a school reported a seroprevalence between 10.4% and 25.86% [24, 43, 44, 49, 58, 62, 130] This large discrepancy could be attributable to the close proximity of care/nursing home residents, shared living spaces and the intimate care and handling of residents by staff.

### Limitations

This systematic review and meta-analysis had several limitations. Firstly, of the 115 studies included in this review, not all of them could be included in sub-analysis as complete data sets could not be retrieved from every study and data quality was heterogeneous. As this review only included studies performed in Europe, the results of this review can only be applied to European countries. By only including papers written in English we may have overlooked important seroprevalence studies conducted in Europe but published in another language. In addition, most studies were performed either in the UK, Italy, Spain or Germany. There was a large gap in studies being performed in Eastern European countries. Those studies performed in the UK predominately took place in the South of England. Therefore, our review maybe bias towards countries and regions where there is more funding and resources available to conduct seroprevalence studies. In this review the studies used a variety of different assays, with different sensitivities and specificities which could lead to an under or overestimation of the true prevalence reported. Furthermore, many of the studies were pre-print articles that had not undergone peer-review; however, study quality was assessed and subgroup analysis based on risk of bias was performed.

## Conclusion

This systematic review and metanalysis highlights substantial heterogeneity between countries, within countries, among professions, and among settings. This heterogeneity, in addition to indicating the general trajectory of the pandemic in different regions, might have been driven by a variety of other factors including governmental policies and restrictions, local guidelines and restrictions, availability of PPE, the time period when the study was conducted and serological test performance. Nevertheless, seroprevalence studies yield large amounts of useful, locally relevant information and should be regularly repeated as the pandemic evolves and local guidelines and restrictions change. As testing standardises and new studies are reported they will also help identify different national experiences across Europe and provide a means to distil best pandemic control practices for the future. As mass vaccinations have been launched in many countries seroprevalence studies will be able to provide valuable information on the immune response over time following vaccination and the need for booster vaccinations, help understand if herd immunity has been achieved and identify populations at risk of transmission and therefore need for vaccination. Finally, as new variants of SARS-CoV-2 now emerge, and many countries prepare for future waves it is vital that regular seroprevalence studies are conducted to aid control by informing public health measures.

## Supporting information

S1 Table Breakdown of quality of assessment results. (XLSX)

S2 Table Commercial assays and their specificity and sensitivity according to the manufacturer. In some cases, the manufacture’s specificity and sensitivity were unable to be found, so evaluation study data was used instead.
(PDF)

S1 Fig Forest plot of the seroprevalence among HCWs stratified by risk of exposure to SARS-CoV-2 patients. (TIFF)
