# Supplementary material for: The seroprevalence of SARS-CoV-2 during the first wave in Europe 2020: A systematic review
Source: PLoS One. 2021 Nov 2;16(11):e0250541. doi: 10.1371/journal.pone.0250541 (PMC8562786; doi:10.1371/journal.pone.0250541)
Supplement: S2 Table — In some cases, the manufacture’s specificity and sensitivity were unable to be found, so evaluation study data was used instead. (PDF) [file pone.0250541.s002.pdf]

Commercial Assay	Study	Manufacturers Specificity	Manufacturers Sensitivity
Chemiluminescent microparticle immunoassay Abbott Diagnostics ARCHITECT SARS-CoV-2 IgG	117,64,67,124,122,106,41,25,50,134,86,120,59,104,112	99.6% (99·0%; 99·9%)	100 % (95·8%; 100%)
SARS-CoV-2 (IgA/IgG) ELISA EUROIMMUN Medizinische Labordiagnostik, Lübeck, Germany	117,80,64,61,56,32,67,54,55,45,65,53,70,27,24,57,30,133,23,132,26,60,52,91,58,62,48	99.6% (IgG) 98,3 %(IgA)	94.4 % (IgG) 96.9%(IgA)
Anti-SARS-CoV-2 NCP ELISA (IgG/IgM) EUROIMMUN Medizinische Labordiagnostik, Lübeck, Germany	45	99.88% (IgG) 98.6 %(IgM)	94.6%(IgG) 88.2 % (IgM)
Chemiluminescence immunoassay Diasorin LIAISON® SARS-CoV-2 S1/S2 IgG Assay)	64,32,87,26,84,121,62,74	99.3 (98·6%; 99·6%)	97.6 (87·4%; 99.6%)
Maglumi 2019-nCoV IgG/IgM fully automated quantitative chemiluminescent immunoassay Snibe, Shenzhen, China	28,85,78,98,71,127,95,88,62	100%(IgG)	91.21%(IgG)
Vircell COVID-19 ELISA (Vircell Spain S.L.U., Granada, Spain)	67,97,26,74,34	99% (IgM+IgA) 98%(IgG)	88% (IgM+IgA). 85%(IgG)
Testsealabs® IgG/IgM Rapid Test Cassette, Hangzhou Testsea Biotechnology Co., Ltd	107	100%(IgG,IgM)	96% (IgG). 88%(IgM)
COVID-19 IgG/IgM rapid test cassette by Zhejiang Orient Gene Biotech Co., Ltd	81,110,113,99,104	100% (95%CI: 93.4% - 100%)	95.8% (95% CI:89.6% - 98.8%)
Screen Test Covid-19 2019-nCOV IgG/IgM by Screen Italia S.r.l	81	IgG: 98.0% (89.4%-99.9%) IgM: 96% (86.3%-99.5%)	IgG:100% (86.0%-100%) IgM: 85% (62.1%-96.8%)
Wantai SARS-CoV-2 Ab Rapid Test Lateral Flow Assay	82	98.8% (93.3%-99.8%)	100% (88.7%-100%)
AFIAS COVID-19; Boditech, Gang-won-do, Korea	83	96.50%	87.50%

VITROS (Ortho Clinical Diagnostics, Rochester, NY, USA)	97	100% (99.1–100.0%)	100.0% (92.7–100.0%) *positive percentage agreement reported
Virusee by Genobio Pharmaceutical, Shanghai, China	93	96.8% (IgG and IgM)	96% (IgG) 94.6% (IgM)
IgM/IgG Antibody to SARS-CoV-2 lateral flow test; Livzon Diagnostics Inc, Zhuhai, Guangdong, China)	36,35,39,101	99.20%	90.60%
POC test: Abbexa (Cambridge, UK) COVID-19 IgG/IgM Rapid Test Kit (abx294171) detecting antibodies against the SARS-CoV-2 nucleocapsid (N) and spike (S)-antigens.	135	97.94%	98.50%
SARS-CoV-2 IgG multiplex particle-based flow cytometry (Luminex) assay developed at Cambridge University Hospital detecting antibodies against the SARS-CoV-2 N and full-length S-antigens1	135	100%	84%
Roche Elecsys AntiSARS-CoV-2 immunoassay (Roche Diagnostics, Rotkreuz, Switzerland)	25,70,129,133,26,69,121,63	99.8% (99·7%; 99·9%)	100% (88·3%; 100%)
ELISA) IgG2 using a SARS-CoV-2 S spike and Nucleocapsid recombinant antigens (Diapro (Palex), Italy)	96		98%
SARS-CoV-2 total Ab ELISA test Wantai Biological Pharmacy Enterprise	41,70,38,23,40,26,90	97.5% (91.3% - 99.3%)	96.7% (83.3% - 99.4%)
COVID-19 IgM anti-N and IgG anti-N) ELISA Assay Kit (EDI/Eagle)	70	100% (IgG) 100% (IgM)	100% (IgG) 45% (IgM)
MP Rapid Test SARS-CoV-2 IgG/IgM (AMP Diagnostics, AMEDA Laboradiagnostik GmbH, Graz, Austria)	33	97.3% (IgM) 96.4% (IgG)	95.7% (IgM) 91.8% (IgG)
Commercial chemiluminesce immunoassay (CLIA), (Medical Systems, 2019-nCoV IgM/IgG, Genova, Italy)	77	For IgG at a specificity of 99.1%. For IgM specificity of 98.99% **According to the study**	For IgG sensitivity was 50%. For IgM sensitivity was 0%. **According to study**

The anti-SARS-CoV-2 (IgG/IgM) POC-test (lateral flow) WONDFO	39	98.7% (97.4-99.4)	94.7% (95% CI: 89.8-97.7)
All Test®2019-nCoV IgG/IgM RapidTest Casette of Hangzhou All Test Biotech Co Ltd	105	98% (IgG) 96%(IgM)	99.9% (IgG) 85%(IgM)
Lysine Lateral Flow Test	101		98.6% **According to the study**
Sure Screen Lateral Flow Test	101	99.67%	96.55%
VivaDiag COVID-19 immunoglobulin (Ig) G/IgM rapid lateral flow qualitative immunoassay, VivaChek Biotech, China)	75	100%	42%
CTK Biotech COVID-19 split IgG/IgM Rapid Test (CTK Biotech, Poway, CA, USA)	120	97.80%	97.10%
Encode SARS-CoV-2 split IgM/IgG One Step Rapid Test Device (Zhuhai Encode Medical Engineering, Zhuhai, China)	120	99·0% [95% CI:94·6–100) **According to the study**	93·4% [95% CI 87·8–96·9] **According to the study**
EDI Novel Coronavirus COVID-19 IgG ELISA kit (Epitope Diagnostics, San Diego, CA, USA	120,62,26	99.8 (99·1%; 99·97%)	98.4 (95·4%; 99·5%)
IgG/IgM Rapid Cassette (Acro Biotech, Inc.,Rancho Cucamonga, CA, USA)	73	IgG: 98.0% (89.4%-99.9%) IgM: 96% (86.3%-99.5%)	IgG:100% (96-100%) IgM:85% (62.1- 96.8%)
Beckman Coulter Access SARS-CoV-2 IgG assay	51	99.8%	100%
POC GenBody COVID-19 IgM/IgG	68	98.8% (93.3% -99.8%)	60% (42.3% -75.4%)
SARS-CoV-2 nucleocapsid protein (NP) ELISA, IgG class antibodies (ImmunoDiagnostics, Hongkong)	26	93.33%	92.50%
SARS-CoV-2 nucleocapsid protein (NP) ELISA, IgM class antibodies (ImmunoDiagnostics, Hongkong)	26		
EDI Novel Coronavirus COVID-19 IgM ELISA kit (Epitope Diagnostics Inc., San Diego, CA	26	100%	

ID Screen ® SARS-CoV-2-N IgG Indirect ELISA (IDvet, Grabels, France; nucleocapsid protein antigen)	26	99.9% (99.6-100%)	93.3% (78.8-98.2%)
Point-of-care Ab diagnostic test, Toda Coronadiag® (TODA Pharma, Strasbourg France)	42		
PCL COVID-19 immunoglobulin (Ig) G/IgM Rapid Gold test (PCL Inc., Seoul, Republic of Korea)	103	100% (94.8%; 100%)	96.7% (83.3%; 99.4%)
Point-of-care lateral flow immunoassay (BioMedomics IgM-IgG Combined Antibody Rapid Test	79	90.63%	88.66%
COVID-19 IgG/IgM Rapid Test Cassette; Multi-G	29	97% **According to the study**	92.2% **According to the study**
Lateral Flow Healgen Scientific	126	97.50%	100%
Luminex based assay of Corona Immunitas	116	99.60%	93.30%
COVID-19 IgG/IgM Rapid Test, Prima Lab, Balerna, CH	72	98% (IgG) 96%(IgM)	100% (IgG) 85% (IgM)
Lateral Flow Assay Fortress Diagnostics	137	98.6% [95% CI: 97.1% to 99.4%]	84.4% [95% CI: 70.5% to 93.5%]
YHLO's iFlash 1800	37	99.30%	94%
REAL COVID19 Rapid test cassette; Durviz, Valencia, Spain; reference RPPCOV1925	105	89.4% – 99.9%	86% – 100%
