# Supplementary figures and images for: The seroprevalence of SARS-CoV-2 during the first wave in Europe 2020: A systematic review
Source: PLoS One. 2021 Nov 2;16(11):e0250541. doi: 10.1371/journal.pone.0250541 (PMC8562786; doi:10.1371/journal.pone.0250541)

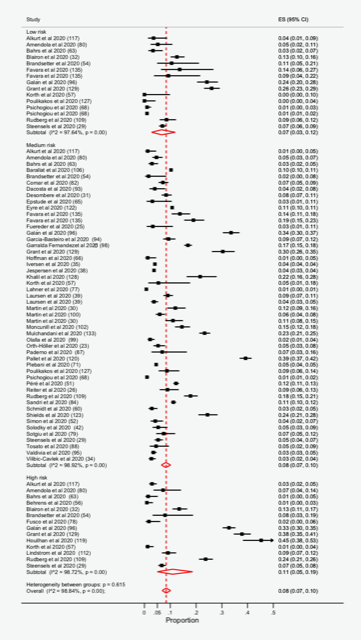

Supplement: S1 Fig — (TIFF) [file pone.0250541.s003.tiff]
